# Supplementary material for: Are carbon emissions trading and green financial instruments synergistic? -Comprehensive quantitative research based on content analysis
Source: PLoS One. 2024 Mar 7;19(3):e0298601. doi: 10.1371/journal.pone.0298601 (PMC10919601; doi:10.1371/journal.pone.0298601)
Supplement: S1 Data — (ZIP) [file pone.0298601.s001.zip › Data/地级市Z_F工作报告.zip!/║■─╧/╒┼╝╥╜τ╩╨/└┤╫╘╣1⁄2╓┌║┼í╛┬φ┐╦╩2╛▌═°í┐_2019.docx]

2018年工作回顾

一年来，在省委、省政府和市委的坚强领导下，我们坚持以习近平新时代中国特色社会主义思想为指导，全面落实新发展理念，深入实施“对标提质、旅游强市”战略和“11567”总体思路，凝心聚力、攻坚克难，扎实推进“六稳”工作，保持了经济社会持续健康发展。

（一）高质量发展态势初步显现。主要经济指标平稳增长。实现地区生产总值580亿元（预计数，下同），增长7.5%；固定资产投资增长10.2%，增速高于全省平均水平；完成同口径一般公共预算收入63.76亿元，增长14.57%；城乡居民人均可支配收入分别达24802元和9596元，分别增长8.1%和10.4%；实现社会消费品零售总额241.57亿元，增长10.2%。财税质量不断提升。一般公共预算收入占地区生产总值比重达到11%，税收收入占地方财政收入比重达到67.11%，非税收入占比下降10.86个百分点。实体经济不断壮大。新增各类市场主体1.69万户，增长15.78%；完成“个转企”393户、“小升规”125户；新增注册商标1776件，增长50.6%。

（二）三大攻坚战成效明显。防范化解重大风险有力有效。出台政府隐性债务防范化解方案和严控地方政府债务十项举措，全面清理核实政府性债务，偿还2018年到期债务51.29亿元；“停缓调撤”项目128个，压减投资总额479.8亿元，其中压减2018年计划投资54.98亿元；抓好平台公司清理整顿和市场化转型，17家平台公司整合压减到10家；成立投融资决策委员会，组建担保集团，加强国库资金监管，做好存量债务置换，全市政府债务风险总体可控。打击处置非法集资，金融稳定发展态势得到巩固。精准脱贫取得新突破。扎实开展脱贫攻坚“春夏秋冬”四场战役，实现6.47万贫困人口脱贫，180个贫困村脱贫出列。解决2.95万贫困人口饮水问题，实施220个村电网改造，完成自然村通水泥路1850公里，易地扶贫搬迁16793人、农村危房改造9088户。电商扶贫、旅游扶贫精准高效；全国乡村旅游暨旅游扶贫工作推进大会在我市召开，旅游扶贫“张家界样本”得到推介。深入实施脱贫攻坚作风建设年，上级反馈交办的突出问题整改到位。污染防治力度空前。持续打好大气、水、土壤污染防治“三大战役”，全面落实河长制，深入开展“绿盾2018”专项行动和“夏季攻势”，强力推进中央和省环保督察问题整改，污染防治取得明显成效。中央环保督察交办71件信访件全部办结、反馈9个问题办结销号7个，解决了一批群众反映强烈的突出环境问题。持续加强生态保护，核心景区移民搬迁加快推进，完成人工造林8万亩。

（三）旅游龙头作用进一步凸显。全年接待游客8521.7万人次、实现旅游总收入756.8亿元，分别增长16.17%和21.33%。全域旅游基地建设成效明显。核心景区空中田园观光游览线顺利开通，九天峰恋旅游区、红二方面军长征出发地纪念馆对外开放，大峡谷、天门山周边环境整治取得实效。文旅融合、农旅融合、体旅融合等新业态不断涌现，张家界千古情、桃花溪谷等项目加快建设，梓山漫居、水木潇湘等精品民宿深受市场青睐，马儿山、红岩岭等乡村旅游景点快速发展，15条旅游线路分别入选武陵山片区房车旅游、大湘西地区文化生态旅游、全省美丽乡村旅游精品线路。旅游国际化步伐加快。成功主办2018丝绸之路工商领导人峰会、东北亚地区地方政府联合会第十二次全体会议、首届芦笋大健康亚洲论坛等国际性会议，世界翼装飞行锦标赛、黄龙音乐季、国际旅游诗歌节等赛事活动成功举行，开通河内、胡志明市、雅加达等国际航线，设立布拉格丝路驿站，张家界国际影响力不断扩大。旅游服务管理水平大幅提升。全国首部全域旅游地方性法规《张家界市全域旅游促进条例》出台施行。全域通系统不断完善，“智慧旅游”提质升级。吴家峪游客服务中心基本建成，新改建旅游厕所137座，邢大、利九等旅游公路建成通车。开展“铁腕治旅”，实施十大重点整治行动，兑现六大承诺，落实六大机制。

（四）产业转型升级不断加快。扎实推进“产业发展年”活动，积极对接省“五个100”项目，完成“52151行动计划”投资85.9亿元。以旅游业为主导的现代产业体系加快形成，产业结构不断优化。工业经济趋稳向好。工业固定资产投资和技改投资分别增长10%、15%；实现工业税收4.69亿元，增长30.84%；新增入规企业17家。“三区一园”加快建设，新增入园企业20家，新开工项目19个，竣工投产18个。张家界经开区获批省级高新区。农业农村加快发展。大鲵养殖达到263.68万尾，商品蔬菜基地、茶园和水果种植面积分别达到34.61万亩、12.92万亩、58万亩。市农业投资公司成功组建。永定区、慈利县获批全国农村三次产业融合发展先导区和试点县。现代服务业水平不断提升。市县两级电商公共服务中心开业运营，电商交易额达55亿元，增长37%。武陵山珍馆、张家界农副产品综合交易大市场建成营业，10个乡镇农贸市场建成使用。金融服务实体经济能力不断增强，11家省级金融机构协议支持1131亿元推动绿色经济发展，市担保集团提供融资担保6.75亿元；金融机构存贷款余额分别增长7.35%、18.75%，新增存款58.5亿元、贷款104.5亿元，存贷比77.5%。

（五）城市品位明显提升。规划体系进一步健全。完成城市总体规划修改和城乡规划技术规定修订，编制了沙堤片区、且住岗组团控制性详细规划和657个村庄规划，三维仿真辅助决策系统建成使用。城市功能进一步增强。黔张常铁路、张吉怀铁路、安慈高速加快建设。武陵山大道、沙堤大道、子午西路延伸段、绕城公路城西段建成通车，且住岗路、大桥路拓宽改造、陈家溪桥等项目竣工使用。8个公共停车场、30座高标准公共厕所建成使用，张家界旅游客运综合枢纽等项目开工建设。云计算大数据中心全面建成。以“六城同创”为抓手，加强城市管理。开建、配建一批限价商品房。加强群众性精神文明建设，省级文明城市创建成效明显。县城和小城镇建设进一步加快。慈利新区路网基本形成，桑植高铁新区加快建设。沅古坪、中湖、阳和、刘家坪等特色小镇魅力初显。乡村振兴战略加快实施，农村“双改”深入推进。

（六）改革开放深入推进。重点领域改革实现新突破。深入推进“放管服”改革，取消、调整行政权力事项53项，清理行政审批中介服务事项77项，新政务服务大厅正式启用，“互联网+政务服务”“最多跑一次”改革取得重要成效。农村承包地确权登记颁证、农村集体资产清产核资基本完成。旅游企业发票门票“一票制”改革全面推开，全域旅游“公对公”结算改革试点推进。财税、文化、司法等领域改革取得实效。创新发展取得新成效。张家界农业科技园成功创建国家农业科技园区，五倍子、大鲵繁育与精深加工技术研究中心获批省级工程技术中心，首个院士专家工作站入驻。降低创业门槛和成本，发放创业担保贷款4600余万元。扩大开放再创新优势。荷花国际机场旅客吞吐量突破220万人次，迈入全国中型机场行列。首家离境退税店挂牌营业，四个进境指定口岸建设进展顺利，公共保税仓、保税商品展示中心建成。招商引资取得新突破，新引进4家500强企业。外贸进出口和利用外资分别增长5.2%、10.7%。

（七）民生福祉持续改善。民生支出占一般公共预算支出比重达78.3%，30件40个省市重点民生实事项目全面完成。民生保障持续加强。新增城镇就业1万余人、农村劳动力转移就业1.3万余人、失业人员再就业3000余人；城乡基础养老金最低标准由85元提高到103元，企业退休人员养老金待遇月人均增加133.06元；残疾人、低保对象等困难群众补助标准进一步提高；医疗保障“一站式”结算服务、乡镇社工站建设走在全省前列。社会事业不断进步。改造41所农村义务教育薄弱学校，完成100所义务教育学校标准化建设，较好完成义务教育“超大班额”化解任务，永定、慈利义务教育均衡发展通过国家督导评估验收。市人民医院新院建设顺利推进，全市公立医疗卫生机构取消药品加成，实施药品集中配送，公立医院分级诊疗人次同比增长10.8倍。计划生育工作实现保类进位。国家卫生城市和食品安全示范城市创建取得阶段性成果。“一宫四中心”建设加快推进。社会大局和谐稳定。化解信访积案40件，中央第八巡视组移交91件信访件全部办结，一批信访突出问题得到妥善化解。持续强化安全生产监管执法，我市被评为全省安全生产工作优秀单位。着力构建天网地网人网“三维一体”的治安防控体系，深入开展扫黑除恶专项斗争，人民群众安全感和满意度不断提升。

(八)政府自身建设全面加强。坚持把习近平新时代中国特色社会主义思想作为政府工作的根本遵循，把全面从严治党贯穿于政府建设始终。政治建设深入推进。依法行政全面推进。行政效能不断提升。廉政建设持续深化。

2019年工作安排

今年政府工作的总体要求是：以习近平新时代中国特色社会主义思想为指导，认真贯彻落实中央、省委和市委经济工作会议精神，坚持稳中求进工作总基调，坚持新发展理念，坚持推动高质量发展，坚持以供给侧结构性改革为主线，坚持深化市场化改革、扩大高水平开放，深入实施“创新引领、开放崛起”战略、“对标提质、旅游强市”战略和“11567”总体思路，着力抓重点、补短板、强弱项，继续打好三大攻坚战，继续开展产业项目建设年活动，扎实开展招商引资项目攻坚年活动，进一步稳就业、稳金融、稳外贸、稳外资、稳投资、稳预期，保持定力、提振信心，迎难而上、担当作为，保持经济持续健康发展和社会大局稳定，奋力迈向优质旅游发展新时代，为全面建成小康社会收官打下决定性基础，以优异成绩庆祝中华人民共和国成立70周年。

今年全市经济社会发展的主要预期目标是：地区生产总值增长8%，规模工业增加值增长7%，固定资产投资增长13%，旅游总收入增长23%，社会消费品零售总额增长10.5%，一般公共预算收入增长10%，城镇居民、农村居民人均可支配收入分别增长9%和11%，城镇登记失业率控制在4.5%以内，居民消费价格指数涨幅控制在4%以内，节能减排、安全生产等完成省定目标。

2019年重点做好十个方面工作

（一）大力推动旅游业高质量发展。按照“三星拱月、月照三星”总体布局、“双核三极多点”产业布局，做精做美全域旅游，争创国家旅游经济示范区。坚持核心景区引领，不断丰富旅游产品供给。突出武陵源核心景区主体地位，发挥主导作用，加快景区提质改造；加强张家界大峡谷环境综合整治，支持创建5A级景区；加快建设天门山现代服务业示范集聚区，做大南线旅游经济圈；围绕红色、户外、水资源做活西线旅游，确保全线开放。建成大庸古城、桃花溪谷、冰雪世界、吃遍中国、红军体验园、张家界千古情等项目，推进复华文旅、武陵古镇等项目建设。着力抓好星级酒店、旅游厕所等服务设施建设，引导重点景区周边片区开发特色客栈。依托重点景区梯次开发、依托特色资源点状开发乡村旅游。推进“旅游+”深度融合。坚持统一打好“张家界”品牌，不断扩大旅游国际影响力。以“张家界”品牌整合资源，强化市场营销。以长三角、珠三角、大中城市等为重点开展客源市场大营销，整合武陵山片区、大湘西联盟、张吉怀等地旅游资源开展联合营销。坚持以外促内，持续加大旅游国际营销，主动加强与国际旅游组织、国际一流旅游目的地的交流合作，讲好张家界故事，传递张家界的好声音；支持旅游企业参加国际旅游展，积极争取国际性会议和体育赛事落户张家界；充分利用友好城市、港澳侨务、旅游智库等资源拓展境内外市场。坚持全域服务提升，不断优化旅游市场环境。深化旅游改革创新，大力推进旅游管理服务标准化、精细化、信息化、国际化，加快“智慧旅游”建设，完善“1+3+N”旅游联合执法体系。加强旅行社规范管理，全面推行电子合同使用、全域旅游结算“公对公”、导游归社工作。深入开展“铁腕治旅”专项整治行动，重拳整治旅游乱象。

（二）着力弥补工业短板。要聚力园区发展，做大总量、提升质量，以更大力度、更有效举措推进新型工业化。一是狠抓工业园区建设。进一步理顺“三区一园”体制机制，推动土地、金融、技术等要素向园区集中，突出抓好园区基础设施及住房、公交、医疗、教育、商业等配套服务设施建设。加大张家界高新区创建国家级高新区工作力度，支持慈利工业集中区创建省级工业园区，加快推进张家界旅游商品产业园建设。二是狠抓产业集聚发展。依托园区，吸纳优势企业、集中上下游企业，加快形成产业链。围绕农产品加工、生物医药、旅游商品加工、信息与高端装备制造“四大工业支柱产业”，加快推进康美大德、昱辰手机、湘丰茶叶等项目建设，扶持久瑞生物、茅岩莓、乾坤生物等企业做大做强。积极谋划智能制造等前沿产业，推动中德零碳科技项目尽快落地。三是狠抓企业培育服务。以企业为主体、市场为导向、大专院校及科研单位为依托，建立健全产学研体系，推动技术创新、模式创新。以“双联”和“三抓三促”活动为载体，加大企业帮扶力度。加强对骨干企业的运行调度，抓好项目签约落地、规模企业培育。

（三）深入实施乡村振兴战略。坚持农业农村优先发展，把乡村振兴作为新时代“三农”工作的总抓手，构建“1+1+6”政策框架体系，强化组织实施，加快实现农业强、农村美、农民富。一是大力发展现代农业。坚持以规模化、特色化、品牌化发展为核心，以工促农、以商活农、以旅带农、科技强农、规模兴农。在稳定粮油总产的基础上，致力发展大鲵、蔬菜、茶叶、水果和中药材“五大农业特色产业”；以桑植县成为省中药材种植基地示范县为契机，建成大湘西中药材市场，培育壮大中医药产业链。大力推进“百企千社万户”现代农业发展工程。实施农产品品牌战略，发展“一县一特”，做强张家界大鲵、张家界莓茶、“硒有慈利”、桑植白茶等区域公用品牌。加快培育一批新型农业经营主体。鼓励工商资本进入现代农业，促进农村一二三产业融合发展。二是加快建设美丽乡村。加大农村基础设施投入力度，完成自然村通水泥路352公里、农村公路窄路加宽300公里，建设村镇供水工程302处，新修和加固堤防28.9公里。持续抓好耕地占补平衡和城乡建设用地增减挂钩，补充耕地3000亩，完成增减挂钩复垦面积7000亩。打好农村人居环境整治首仗，重点做好垃圾污水处理、厕所革命、村容村貌提升。三是深化农村综合改革。加快推进农村集体产权制度、“三权分置”和供销合作社改革，完善农村宅基地、土地征收等制度。推进治理能力现代化，健全自治、法治、德治相结合的乡村治理体系。强化农业社会化服务，着力培养一支懂农业、爱农村、爱农民的“三农”工作队伍。创新农村金融服务，推动各类政策、项目、资金向强农富农领域集聚。加快农产品电商平台建设。

（四）培育壮大现代服务业。着力构建电子商务、商贸物流、现代金融、会议会展、健康养老“五大服务产业”,形成多点支撑、多业融合的现代服务业发展格局。加快发展电子商务。继续实施农村电商“个十百千万”工程，着力培育电商示范企业，全面完成慈利、桑植国家电子商务进农村综合示范县建设。加快发展商贸物流业。抓好专业市场建设，加快奇峰市场、义乌国际商贸城二期、农产品批发市场建设，新建7个乡镇农贸市场。完善仓储、冷链物流基础设施。大力发展“互联网+”物流新业态、新模式。加快发展现代金融。大力创新金融产品，积极引进金融机构，加强融资担保体系建设，完善“政银保担投”金融链条，健全信贷风险缓释机制，推进基金、保险、证券、小额贷款等金融业态发展。加快组建桑植村镇银行。加快发展会议会展经济。打造地方会展品牌，引进高层次展会。办好丝绸之路友好城市协作体年会、第十九届国际森林保护节、第三届天门山国际越野赛和亚洲捷兔慢跑大会。加快培育健康养老产业。发挥森林康养、中医药养生、温泉疗养等资源优势，打造“旅游+”健康养老服务产业链。培育发展总部经济、信息经济，促进家政服务、文化体育等服务消费提质扩容。

（五）坚决打好“三大攻坚战”。坚持把打好“三大攻坚战”作为决胜全面小康的重中之重，针对突出问题，打好重点战役。一是坚决打好防范化解重大风险攻坚战。认真落实严控地方政府债务十项举措，强化政府性债务管控，稳妥处理地方政府债务风险，做到坚定、可控、有序、适度。进一步规范PPP与政府购买服务项目，严格执行负面清单管理。加大政策支持力度，积极稳妥推进融资平台市场化转型。广泛开辟财源，拓展收入渠道。树立过“紧日子”思想，做好“三保”资金兜底保障。加快推进财政预算绩效管理，不断提高资金使用效益。切实防范化解扶贫小额信贷风险。二是坚决打赢打好精准脱贫攻坚战。全面对标看齐习近平总书记精准扶贫工作重要论述，向脱贫攻坚最后堡垒发起总攻。以桑植县为主战场，深入实施脱贫攻坚“1332”行动计划，确保桑植县脱贫摘帽，所有贫困村出列，全市贫困发生率降至0.4%以下。要一鼓作气，重点解决好实现“两不愁三保障”面临的突出问题，层层压实责任，加强和规范驻村帮扶结对帮扶工作，抓好产业扶贫、农村危房改造、健康扶贫、电商扶贫、教育扶贫、兜底保障等重点工作，着力激发贫困群众内生动力。统筹解决好贫困村和非贫困村工作不平衡的问题，巩固脱贫成果。三是坚决打好污染防治攻坚战。以高度的政治自觉落实“共抓大保护、不搞大开发”要求，大力加强环境整治，持续开展碧水守护、蓝天保卫、净土攻坚、大地增绿、城乡洁净“五大行动”，持续推进农村建房、集镇环境、旅游干线环境、环境整治“6+1”四大专项整治行动，加强河长制、路长制工作。加大环保问题整改力度，确保完成时序进度目标任务。加强自然保护地管理，全面落实森林防火、松材线虫病等林业有害生物防治工作。深化国家生态文明建设示范市创建。

（六）全力推进重点项目建设。牢牢扭住项目建设“牛鼻子”，扎实推进“产业项目建设年”。一是加强项目谋划。坚持基础设施量力而行、民生项目尽力而为、产业投资全力以赴，注重精算平衡，切实抓好138个重点项目建设，完成投资200亿元。二是加快项目建设。紧扣“五个100”项目，扎实推进“52151行动计划”。建成黔张常铁路，加快张吉怀铁路、安慈高速建设和荷花国际机场配套建设，力争桑龙高速开工，积极做好张石铁路、张官高速、炉慈高速等重大项目前期工作，启动宜冲桥至张清公路连接线前期工作。推进丝路荷花国际文化旅游城、宁邦国际广场等重大产业项目建设，推动市人民医院新院、综合档案馆、垃圾焚烧发电、核心景区移民建镇等社会发展和民生项目建设。三是强化要素保障。积极争取金融机构支持，创新融资模式，充分发挥担保集团作用，提升金融服务水平。抓好土地储备和闲置土地处置，依法推进征地拆迁，保障项目建设用地。严格落实重点项目联席会议等工作机制，完善重点项目建设考核奖励办法。从严打击强揽工程、恶意阻工等违法行为，优化项目建设环境。

（七）致力打造国际精品旅游城市。坚持用国际化理念规划城市、国际化标准建设城市、国际化规则管理城市，以“双修双改”为总抓手，推进国际精品旅游城市建设。一是加强城乡规划管控。全面推进新一轮城市总体规划编制、“多规合一”信息平台建设。完成武陵源风景名胜区总体规划修编和枫香岗组团、荷花组团等控制性详细规划修订，严格落实房地产开发项目公共服务设施配建要求。强化规划批后管理，严控临街退让、临水退让、临山退让，让城市显山露水。加强村镇规划建设管理，推动农村建筑风貌协调、彰显特色、安全宜居。严厉整治城乡违章建筑。二是加大中心城区建管力度。建成杨家界大道、大庸桥立交桥、月亮湾中西路、融山西路、开明路、张罗公路一期及红壁岩大桥、茅溪河大桥，着力推进荷花滨河路及枫香岗大桥、澄潭大桥等项目建设，开工建设五子坡带状公园、樟树角公园二期、仙人溪和黄沙泉湿地公园。加快建设大湘西天然气长输管网和城市集中供气设施，改扩建城市截污管网5.8公里。加快推进智能公共停车场、充电站、垃圾站厕、地下人行通道等项目建设。改造棚户区6091户87.58万平方米。下大力气抓好城市环境综合整治，重拳整治交通秩序、市容市貌，推进物业管理标准化、规范化，加强出租车、网约车应用管理，提升城市宜居宜游宜业水平。优先推进“智慧城市”建设，加快智慧交通、智慧城管、智慧教育、智慧医疗和天网工程建设。持续抓好“六城同创”。三是加快县城和小城镇建设。支持慈利撤县设市。完善慈利县城新区、桑植高铁新区和零溪、三官寺、利福塔、茅岩河等中心镇综合承载功能，加快推进乡镇“四项标配”建设。依托产业特点、人文底蕴和生态禀赋，坚持差异化发展、特色化培育，努力打造国家级、省级特色小镇。

（八）全面深化改革开放。要用好改革开放这个关键一招，聚神聚力抓好改革落实，着力打造武陵山片区对外开放新高地。一是扎实推进各项改革。坚持以供给侧结构性改革为主线不动摇，更多采取改革的办法，更多运用市场化、法治化手段，巩固“三去一降一补”成果，增强微观主体活力，提升产业链水平，畅通国民经济循环。深入推进“放管服”改革，推动“互联网+政务服务”向乡村延伸，实现90%以上政务服务事项“最多跑一次”。深化财税体制改革，持续释放减税降费改革红利。深化商事制度改革，进一步放宽市场准入，加强“双随机一公开”监管，实行“证照分离”“证照联办”。全面完成政府机构改革、武陵源“区局合一”改革。深化国企改革，大力发展混合所有制经济。统筹推进文化、教育、卫生、司法等领域改革。二是着力提升招商引资水平。以“招商引资项目攻坚年”为抓手，努力在产业项目招商、重大招商项目落地上实现新突破。按照国家产业政策导向，策划包装一批大项目、好项目。实施五大对接行动，组团参加“港洽周”“湘商大会”活动。完善“统分结合、归口管理”和重大项目“一事一议”推进机制，确保项目履约率达到70%以上。三是大力推进创新创业。加快培育企业核心竞争力，建设5个创业孵化基地。引导全社会加大研发经费投入，力争全社会研发经费支出占地区生产总值比重达0.75%；推动科技成果转化，培育高新技术企业达到45家以上。推进“质量强市”，注重商标培育，扶持品牌发展。四是支持民营经济发展壮大。坚持“两个毫不动摇”，促进多种所有制经济共同发展。落实省委省政府促进民营经济高质量发展的25条政策措施，切实减税降费，改进融资服务，优化营商环境，激发中小企业发展活力，发挥民营经济在吸纳就业、创造税收、促进发展等方面的重要作用。积极引导成立行业协会、异地商会。五是加快构建开放新格局。引导和鼓励企业开拓境外市场。开展自贸区先行先试工作，完善口岸功能，推进临空产业园、航空物流园规划建设，建成运营指定口岸；加快保税仓、免税店、离境退税店建设，优化口岸通关环境，发展跨境电商等新业态。

（九）着力保障和改善民生。要多谋民生之利，多解民生之忧，让发展更有“温度”。竭诚办好惠民实事。实施好省市30件重点民生实事项目，加强资金和项目调度，严把规划设计关、施工质量关、项目验收关和工程管护关，全力打造精品实事工程。大力繁荣社会事业。落实教育优先发展战略，促进各类教育均衡发展。启动慈利芙蓉学校建设，加快建设雨露学校、丽景小学，建成桑植芙蓉学校、桑植金海实验学校，完成省定大班额化解任务；抓好张家界旅游学校建设，支持张家界市高级技工学校筹建湖南旅游技师学院，加快吉首大学张家界学院转设工作；努力改善教师待遇，加强教师队伍建设。推动文化体育事业加快发展。加快建设贺龙体育中心，建成市“一宫四中心”，大力推进国家公共文化服务体系示范项目创建，实施百村有线电视通村联网工程，打造20个民族民俗文化建设示范点、20个全民健身示范村，办好市第六届全民运动会。加快推进“健康张家界”建设。实施医疗卫生城乡一体化改革，加快推进医联体建设，加强全科医生队伍建设，做实做优家庭医生签约服务；大力开展国家卫生城市创建和爱国卫生运动；进一步优化计划生育服务管理。加强军民融合。着力强化社会保障。抓好高校毕业生、退役军人、农民工、贫困劳动力等重点群体就业创业，确保零就业家庭动态清零。不断扩大社会保险覆盖面，提升保障服务能力和社保待遇水平。健全社会救助体系，提高城乡低保、特困供养标准。注重慰老、爱幼、扶弱、济困，发展社会救助、慈善和残疾人事业，积极推进殡葬改革。强化食品安全综合治理。加强和创新社会治理。完善社会矛盾纠纷多元化解机制。全面加强应急能力建设。完善社会治安防控体系。

（十）切实加强政府自身建设。始终以人民满意为目标，不断增强政府执行力和公信力。坚定政治信仰。用习近平新时代中国特色社会主义思想统筹政府工作，树牢“四个意识”、坚定“四个自信”，践行“两个维护”，严守政治纪律和政治规矩，在政治立场、政治方向、政治原则、政治道路上同以习近平同志为核心的党中央保持高度一致。坚持依法行政。把法治思维贯穿政府工作全过程，自觉带头尊法学法守法用法，推进公共法律服务体系和社会信用体系建设。强化使命担当。加快政府职能转变，不断增强质量意识、创新意识、市场意识、风险意识。加强学习和调查研究，不断增强工作本领，推动工作创造性贯彻落实。筑牢廉政底线。全面加强政府系统作风建设，严格执行廉洁从政各项规定，严格落实党风廉政建设主体责任和“一岗双责”。从源头上预防腐败，以清廉的政风赢得群众的信任和支持。
